# P97 - Characteristic of protein sensibilisation in infants

**DOI:** 10.1186/2045-7022-4-S1-P152

**Published:** 2014-02-28

**Authors:** Tatyana Sentsova, Vera Revyakina, Svetlana Denisova, Olga Monosova, Ilya Vorozhko, Marina Belitskaya, Anna Thimopheeva

**Affiliations:** 1 Department of Allergology, Research Institute of Nutrition RAMS, Moscow, Russian Federation

## Background

Cow milk protein sensibilisation is common in bottle-fed infants. It is known about immunological cross-reaction for cow and goat milk protein.

## Methods

We examined 85 infants (39 girls, 46 boys), 1.5 – 18 months old, who were fed by artificial milk formulas. Gastrointestinal symptoms of food allergy were diagnosed in 45 (52.9%) children with diarrhea and in 40 (47.1%) children with constipation. The control group consisted of 25 healthy infants of the same age. The level of total IgE, cow and goat milk proteins IgE allergenspecific antibodys in coprofiltrates were measured by immunoenzymometric method using the spectrophotometer “Sunrise” (Belgium), with test-systems “Allergopharma” and “Dr. Fooke” (Germany).

## Results

The highest rate of sensibilisation to cow milk protein (89%) was found in infants of 1.5-6.0 months old compared to infants of 6.5-12.0 (75%) and 12.5-18 months old (56%). Increased level of total IgE in coprofiltrates was more common in infants of 1.5-5.5 months old, too, compared to infants of other ages (33%, 25% and 22%, respectively). The sensibilisation to goat milk protein was more common in infants of 6.5-12 months old (59%) and 12.5-18.0 months old (45%) compared to younger (1.5-6.0 months old) infants (23%). In infants of any ages sensibilisation to cow milk protein was more frequent than to goat milk proteins.

## Conclusion

Increasing rate of sensiblisation to goat milk protein in bottle-fed infants more than 6 months old, probably, depends on immunological cross-reaction for cow milk protein.

